# Graphite Carbon-Supported Mo_2_C Nanocomposites by a Single-Step Solid State Reaction for Electrochemical Oxygen Reduction

**DOI:** 10.1371/journal.pone.0138330

**Published:** 2015-09-18

**Authors:** K. Huang, K. Bi, C. Liang, S. Lin, W. J. Wang, T. Z. Yang, J. Liu, R. Zhang, D. Y. Fan, Y. G. Wang, M. Lei

**Affiliations:** 1 State Key Laboratory of Information Photonics and Optical Communications & School of Science, Beijing University of Posts and Telecommunications, Beijing, China; 2 Beijing National Laboratory for Condensed Matter Physics, Institute of Physics, Chinese Academy of Sciences, Beijing, China; 3 School of Materials Science and Engineering, Central South University, Changsha, Hunan, China; University of Edinburgh, UNITED KINGDOM

## Abstract

Novel graphite-molybdenum carbide nanocomposites (G-Mo_2_C) are synthesized by a typical solid state reaction with melamine and MoO_3_ as precursors under inert atmosphere. The characterization results indicate that G-Mo_2_C composites are composed of high crystallization and purity of Mo_2_C and few layers of graphite carbon. Mo_2_C nanoparticles with sizes ranging from 5 to 50 nm are uniformly supported by surrounding graphite layers. It is believed that Mo atom resulting from the reduction of MoO_3_ is beneficial to the immobilization of graphite carbon. Moreover, the electrocatalytic performances of G-Mo_2_C for ORR in alkaline medium are investigated by cyclic voltammetry (CV), rotating disk electrode (RDE) and chronoamperometry test with 3M methanol. The results show that G-Mo_2_C has a considerable catalytic activity and superior methanol tolerance performance for the oxygen reduction reaction (ORR) benefiting from the chemical interaction between the carbide nanoparticles and graphite carbon.

## Introduction

As is well known to all, ORR is the main performance-limiting factor due to its sluggish kinetics in the high-efficiency energy conversion devices such as fuel cells and metal-air batteries [1–15]. To accelerate the ORR process, precious Pt-based electrocatalysts are highly desired, but the limited reserve and increasing price of Pt have been turned out to be great restrictions of such devices [16–18]. Fortunately, due to the recent advancements in anion-exchange membrane materials [19–22], the serious CO_2_-poisoning problem to KOH electrolyte which will reduce the ionic conductivity of the electrolyte and block the pores in the electrode has been overcome. In addition, considering the superior kinetics of the ORR in alkaline solution to that in acidic media, a much wider range of less expensive materials can be used as efficient and stable ORR catalysts in alkaline solution [23–25].

On the other hand, transition metal carbides such as molybdenum carbide and tungsten carbide owning the properties of covalent solids, ionic crystals and transition metals have been proved to own similar electronic and catalytic performances to Pt-group noble metals in reactions such as hydrogenation, dehydrogenation and isomerization of hydrocarbons [26–28]. Recently, Wan et al. synthesized multiple phases of molybdenum carbide as electrocatalysts for hydrogen evolution reaction (HER) and showed promise as an alternative to Pt [29]. Ma et al. modified molybdenum carbide by nickel using a temperature-programmed reaction process and used it for steam reforming of methanol as enhanced catalysts [30]. Yan and coworkers loaded Pt on Mo_2_C particles through ionic exchange process with a synergistic effect and strong interaction force for methanol electro-oxidation [31]. Jager et al. synthesized micro/mesoporous carbide derived carbon powder from Mo_2_C using high-temperature chlorination method as a very active catalyst for ORR [32]. Moreover, Stottlemyer and co-workers have demonstrated that transition metal carbides (TMCs) are well suited materials for the electro/-catalysis of oxygen-containing species due to the strong oxygen-carbide interaction [33]. Thus, in recent years, Liao et al. developed a facile calcination method for novel nanoporous molybdenum carbide wires as an active electrocatalyst towards ORR [34].

However, very limited electrocatalytic studies on the in situ formation of β-Mo_2_C nanocomposite on graphite carbon using organic amines as reductant and carbon source have been investigated as far as we know. And compared with halides used in rapid solid-state method for the synthesis of carbides previously, oxides are cheaper and more stable in nature. Herein, we successfully prepared β-Mo_2_C nanocomposites supported on graphite layers (G-Mo_2_C) with the size of Mo_2_C nanoparticles ranging from ca. 5 nm to 50 nm, which exhibit considerable activity and superior methanol tolerance during ORR process in alkaline electrolyte. And it is believed that the surrounding graphitic layers works as a protective film on the surface of Mo_2_C nanoparticles from being passivated during the ORR operation.

## Experimental

**Fig 1:**
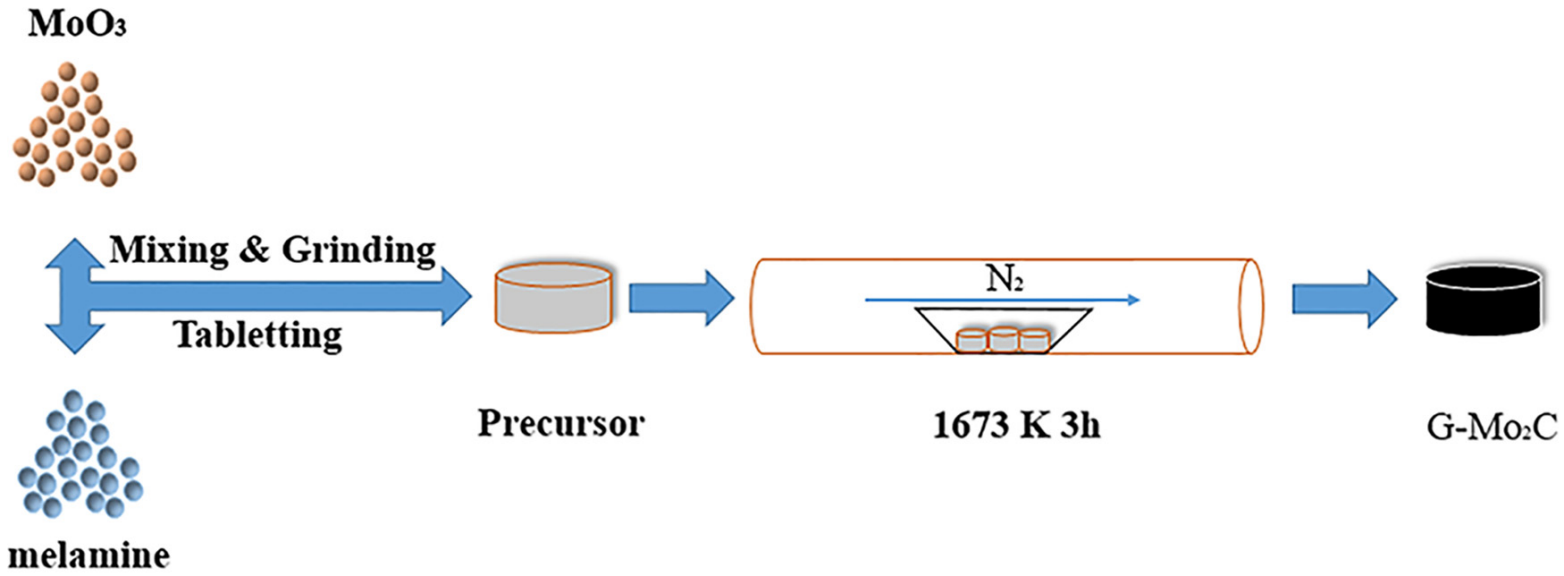
Typical synthesis process of G-Mo_2_C nanocomposites.

All starting materials are of analytical pure grade and arepurchased from commercial sources. Fig 1 shows the typical synthesis process optimized from our previous work beyond the restrictions of evacuating and sealing [35], 0.012 mol melamine and 0.04 mol MoO_3_ powder were mixed together, pressed to a pellet and put in an alumina boat. Then, the alumina boat was placed in the center of a horizontal alumina tubular furnace and flushed with nitrogen atmosphere to remove the remaining air in the alumina tube, the furnace temperature was rapidly increased to 1673 K and kept at the peak temperature for 3 hours under N_2_ flow at 200 sccm. After the furnace was rapidly cooled to the room temperature in the flow of N_2_ atmosphere, the black product was collected from the alumina boat.

Morphology and microstructure of the samples were characterized by TEM (CM200-FEG, Philips), XRD (D/MX-IIIA, RIGAKU) and Raman spectroscopy (INVIA, RENISHAW). Electrochemical characterizations were performed on a CHI660E electrochemical workstation with a three-electrode system consisting of a glassy carbon electrode (5 mm in diameter) loaded with catalysts of 0.5 mg cm^-2^ as the working electrode, a Pt foil as the counter electrode and an Hg/HgO electrode as the reference electrode which was calibrated with respect to reversible hydrogen electrode (RHE) by E_(RHE)_ = E_(Hg/HgO)_ + 0.92 V. CV measurements were performed from 0.1 to 0.8 V with a scan rate of 50 mVs^-1^ in N_2_- and O_2_-saturated 0.1M KOH solution, respectively. Rotating disk electrode (RDE) measurements were conducted at different rotating speed from 400 to 2000 rpm at a scan rate of 5 mv s^-1^ and chronoamperometric responses were carried out at a constant voltage of 0.65 V in O_2_-saturated 0.1M KOH solution adding 3M methanol at 500 s, successively.

## Results and Discussion

**Fig 2:**
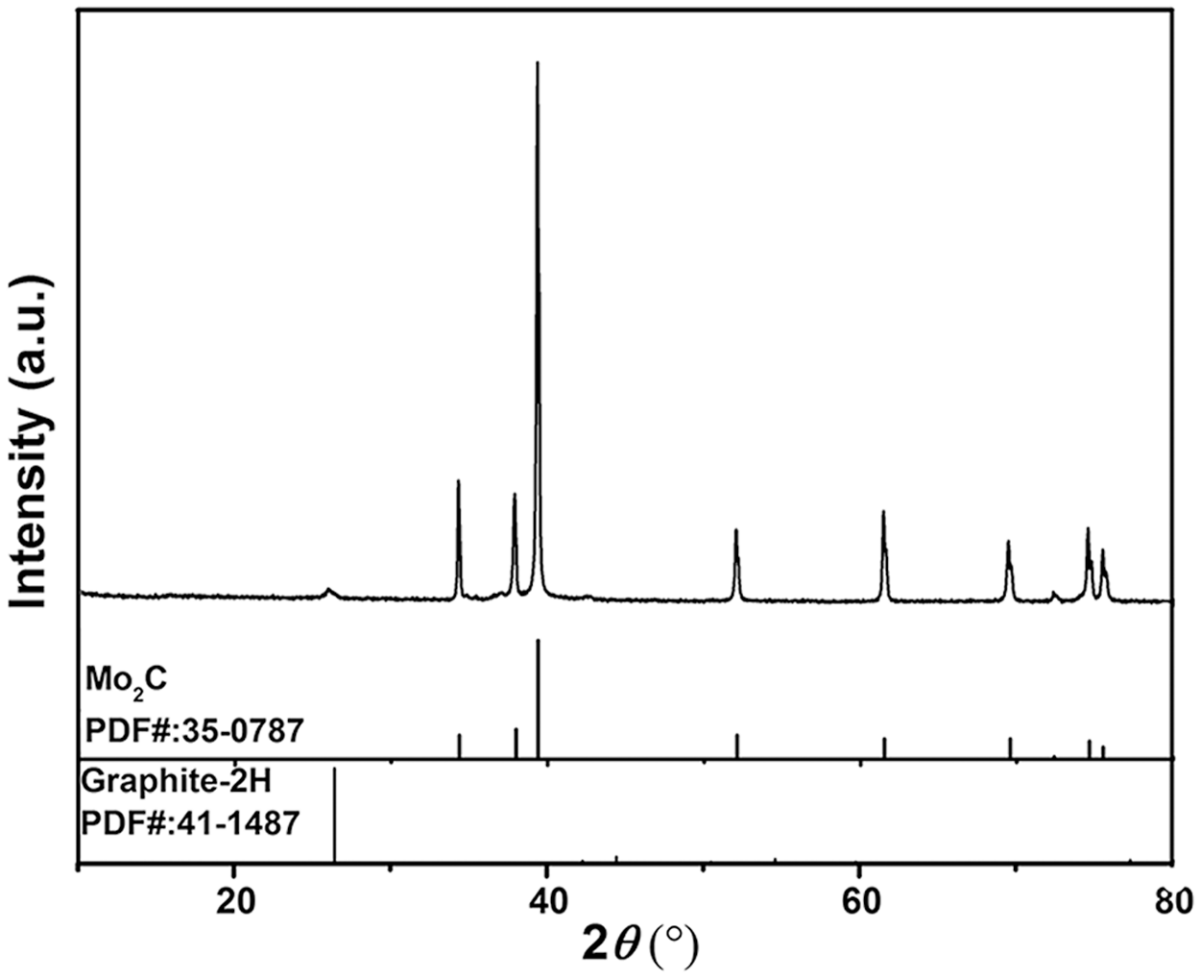
XRD Pattern of G-Mo_2_C with the standard diffraction patterns of Graphite and Mo_2_C.

As shown in Fig 2, the XRD patterns of the synthesized G-Mo_2_C can be typically indexed as the hexagonal-close-packed structure of β-Mo_2_C (JCPDS No. 35–0787) together with graphite-2H carbon (JCPDS No. 35–0787). The sharp and strong peaks and highly exposed (101) plane indicate the good crystallization of the Mo_2_C nanoparticles while the unobvious diffraction peaks of graphite carbon in the pattern can be attribute to the limited content. It is worth pointing out that the Mo:C ratio of 1:1 and 2:1 in molybdenum carbide crystals are the most stable ones with respect to the stoichiometries. And as for Mo_2_C, hexagonal Mo_2_C or β-Mo_2_C is the high-temperature stable phase with a disordered L’3 structure compared with the low-temperature phase of orthorhombic Mo_2_C or α-Mo_2_C which adopts an ordered ξ-Fe_2_N type structure.

**Fig 3:**
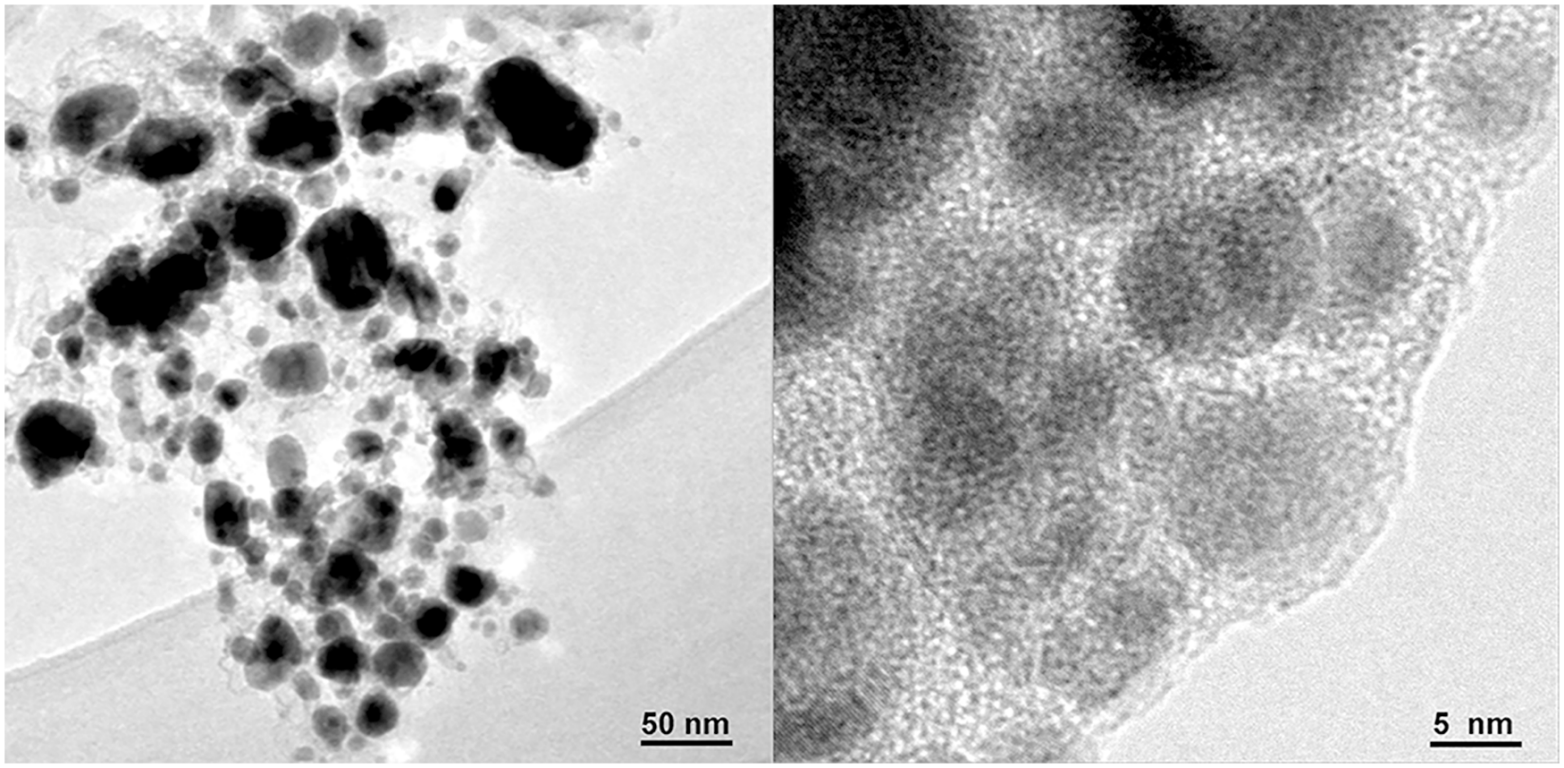
TEM images of G-Mo_2_C at different magnifications.

**Fig 4:**
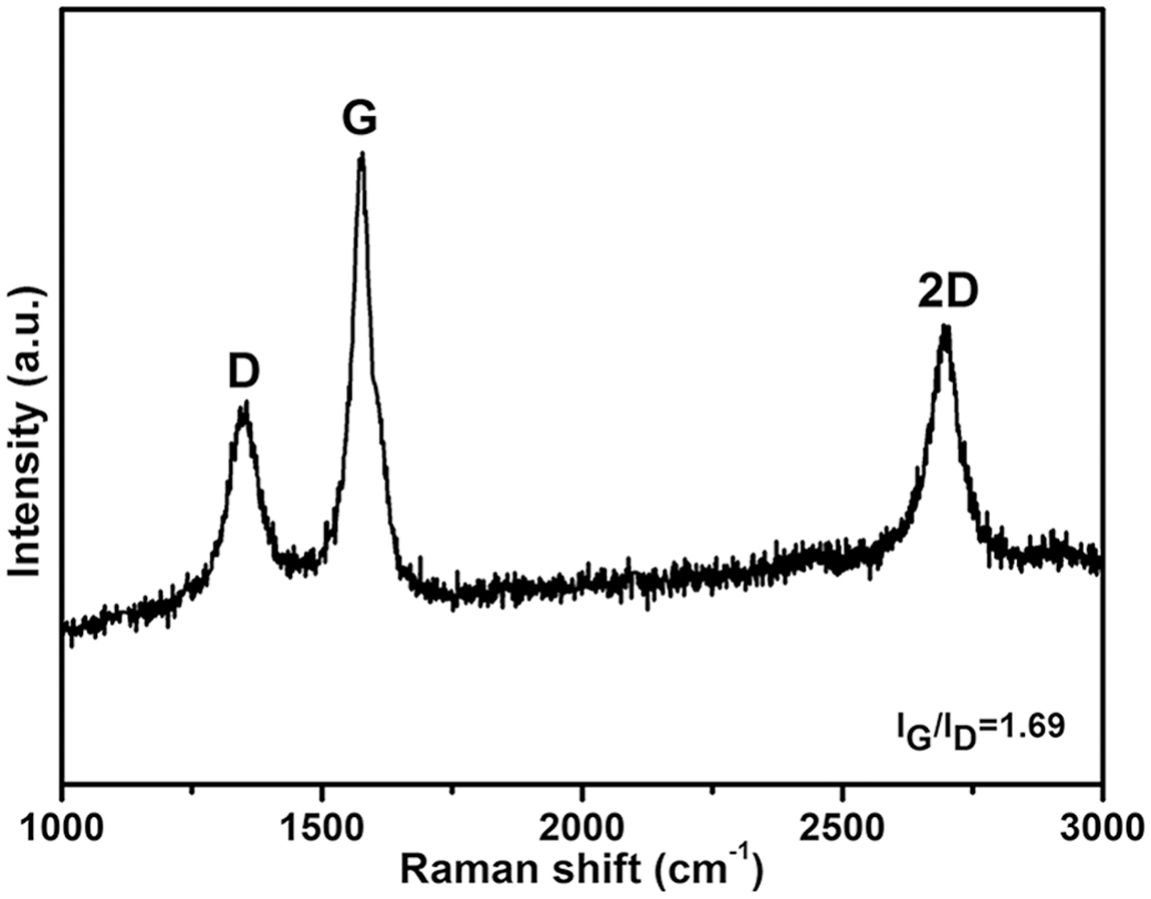
Raman spectrum of graphite in the G-Mo_2_C composite.

To further investigate the configuration of this novel G-Mo_2_C composite, high-resolution transmission electron microscopy (HRTEM) images at different magnification are shown in Fig 3. It can be observed that Mo_2_C nanoparticles with sizes ranging from ca. 5 nm to 50 nm anchored on the layers of graphite carbon uniformly corresponding to the results of XRD analysis. Moreover, since the Raman spectroscopy is a powerful and widely used method for the characterization of graphitization degree of carbon-based materials, Raman spectrum of the G-Mo_2_C is shown in Fig 4, exhibiting three obvious Raman peaks located at 1355, 1579 and 2695 cm^-1^ which can be attributed to the disorder induced D-band, G-band and 2D-band of crystalline graphite respectively and indicate the existence of the ordered graphitic domains in the G-Mo_2_C composites. In addition, the ratio of the G-band to D-band with a value of 1.69 could also be used to judge the high degree of graphitization intuitively [36]. Considering the good contact between graphite carbon layers and β-Mo_2_C nanoparticles, the enhanced overall electronic conductivity can be expected to benefit from the functional graphite carbon matrix.

**Fig 5:**
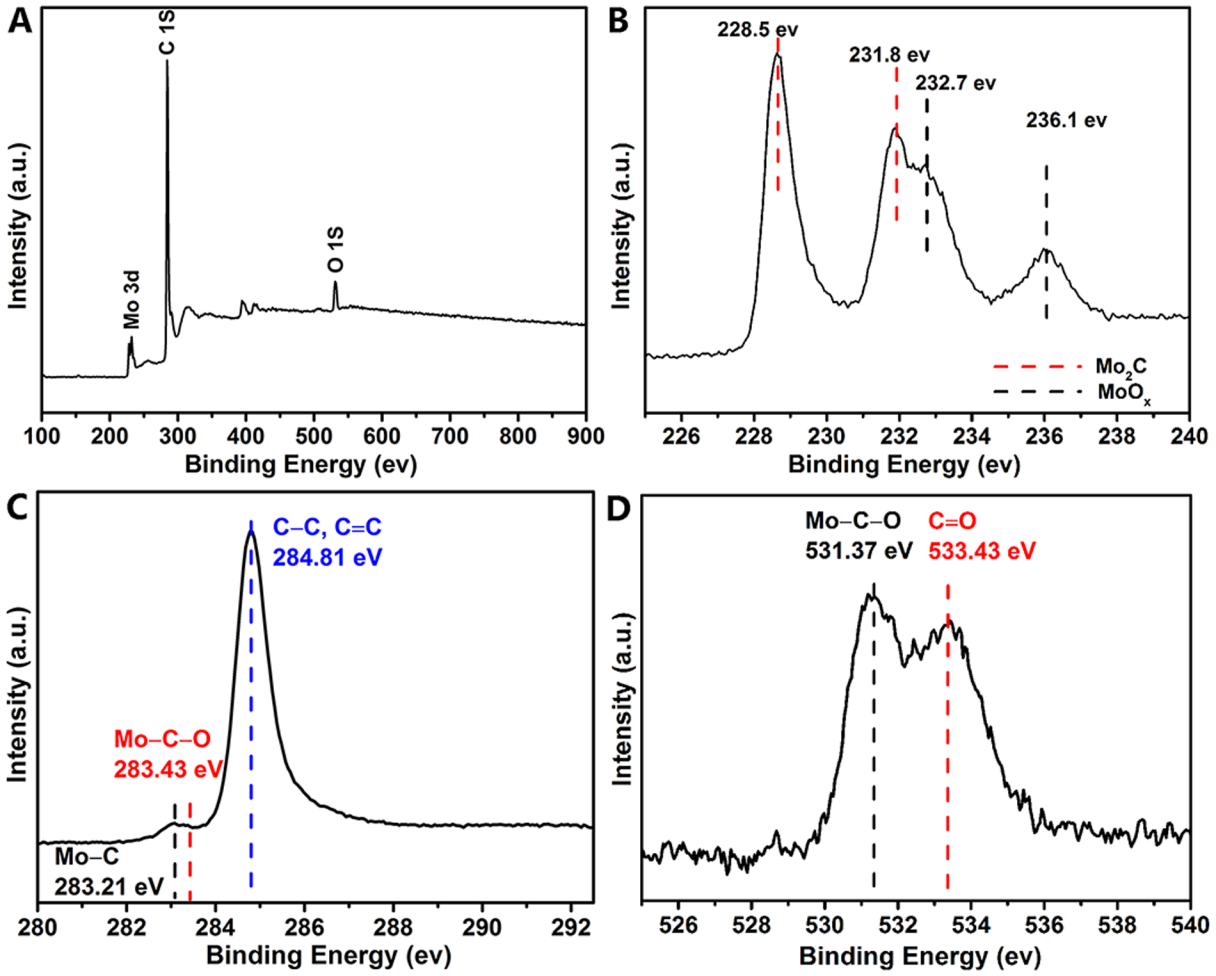
XPS spectra of G-Mo_2_C (A)survey; (B) Mo 3d; (C) C 1s and (D) O 1s.

It is reported that the pyrolysis of melamine will first generate NH_3_ with a series of intermediate condensed phases such as so-called melam ((C_3_N_3_)_2_(NH_2_)_4_(NH)), melem (C_6_N_7_(NH_2_)_3_), melon ((C_6_N_7_)_3_(NH_2_)_3_(NH)_3_), graphitic carbon nitride materials (g-C_3_N_4_), and then further release some chemically reactive hydrogen-, carbon-, and nitrogen-containing atomic species such as C_3_N_3_^+^, C_2_N_2_^+^, C_3_N_2_^+^ and CN_2_H^+^ at higher temperatures [37–40]. Thus, the overall pathway of synthesizing G-Mo_2_C can be concluded as follows: MoO_3_ is reduced into Mo element by the chemically reactive atomic species with possible low-temperature intermediate phase of Mo_x_N, then the as-reduced Mo element is further converted to Mo_2_C with the increasing of temperature [35]. Moreover, controlled test of pyrolysis of melamine tablets at same condition was found to obtain no products in the alumina boat, which suggests that the dangling bonds on Mo atom are beneficial to the immobilization of graphite carbon. To further illuminate the chemical interaction between the Mo_2_C and graphite carbon, the XPS data of G-Mo_2_C in Fig 5 have been provided in consideration of the surface consumption of oxygen on an ORR catalyst. The appearance of surface O species can be ascribed to lattice oxygen in MoO_x_ due to the ageing process in air [41], and the XPS peaks corresponding to Mo-C-O bond in C 1S and O1S spectra directly prove the chemical interaction [42].

**Fig 6:**
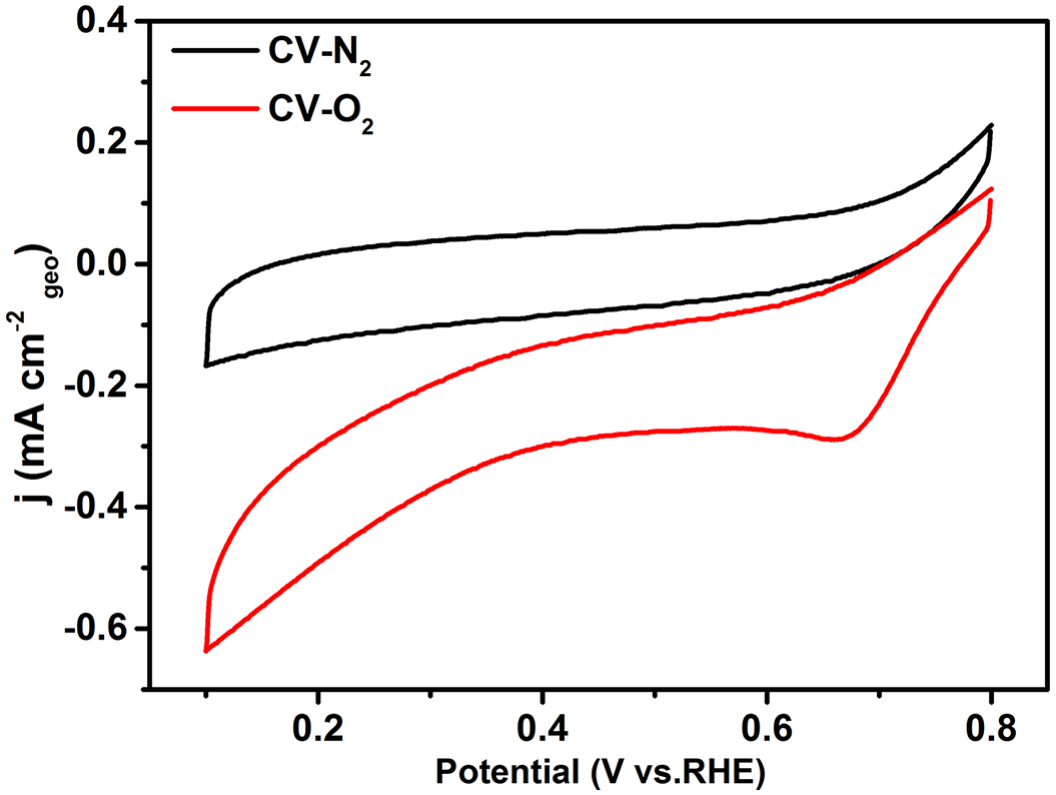
Cyclic voltammetry curves of G-Mo_2_C as ORR catalyst in N_2_-saturated and O_2_-saturated 0.1 M KOH.

**Fig 7:**
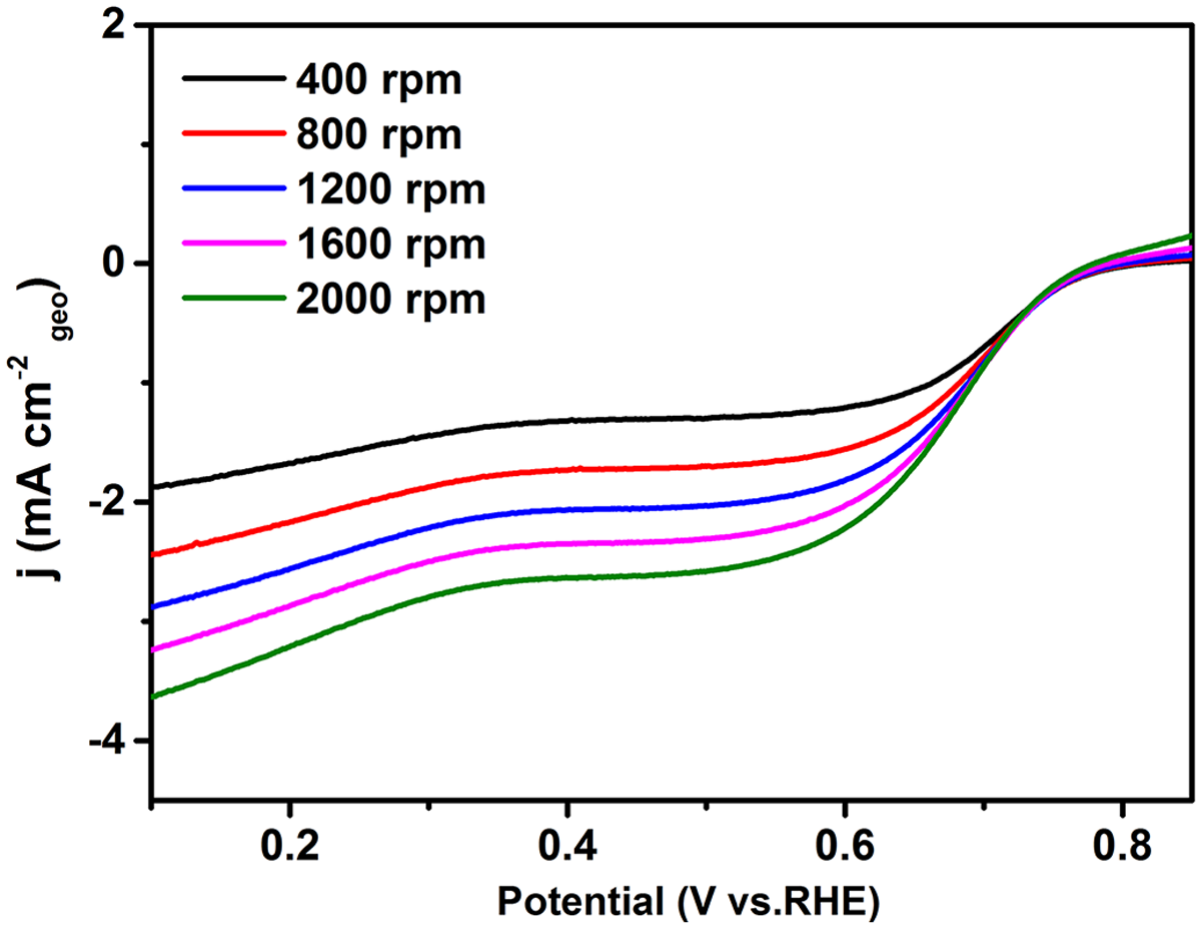
Linear sweep voltammogram of G-Mo_2_C as ORR catalysts in O_2_-saturated 0.1M KOH, Scan rate: 5 mV s^-1^.

In order to investigate the electrocatalytic performance of the G-Mo_2_C composites as a catalyst for the ORR process, CV and RDE methods were employed in 0.1M KOH solution at room temperature. As shown in Fig 6, G-Mo_2_C electrode is featureless in the N_2_-saturated KOH solution during the CV operation, however obvious ORR peak turns up in O_2_-saturated condition suggesting G-Mo_2_C as a suitable catalyst for ORR to some extent. Moreover, the ORR activity of G-Mo_2_C upon mass transfer was further investigated by the RDE measurements with a fixed potential scan rate of 5 mV s^-1^ by increasing the rotation rate from 400 to 2000 rpm as shown in Fig 7. It is clear that the onset potential is about 0.75 V vs RHE and the diffusion current densities are enhanced with the increase of the rotation rate, and the current density at 1600 rpm is about 3.32 mA cm^-2^ at the potential value of 0.1 V. Obviously, there are two reduction peaks at 0.62 V and 0.54 V instead of a steady diffusion current density, indicating a two steps ORR mechanism for G-Mo_2_C.

It is well known to all, there are two major pathways for the reduction of oxygen in alkaline aqueous solution: direct 4-electron pathway to H_2_O and 2-electron pathway with “peroxide” as the reduction product. Thus, ORR kinetics determined by the transferred electron number with G-Mo_2_C as the catalyst are further studied using Koutecky-Levich (K-L) plots according to the parameters reported by Wang and Jirkovsky [43,44]:

$$\frac{1}{J}\;=\;\frac{1}{J_{K}}+\frac{1}{J_{L}}\;=\;\frac{1}{J_{K}}+\frac{1}{B\omega^{\frac{1}{2}}\;}$$ (1)

$$B\;=\;0.2nFCD^{\;\frac{2}{3}}\nu^{-\;\frac{1}{6}}$$ (2)

**Fig 8:**
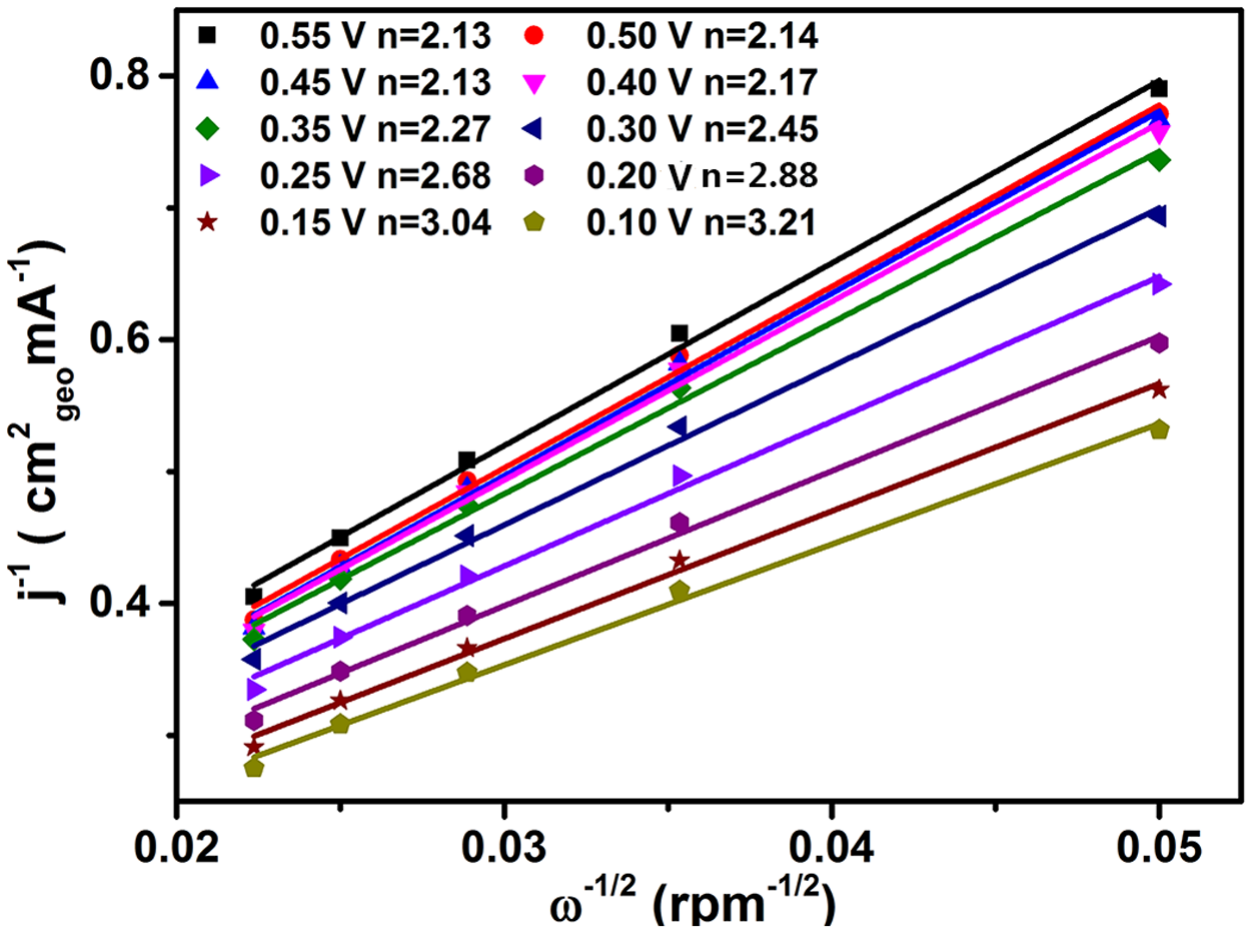
Corresponding K-L plots at different potentials for G-Mo_2_C electrode.

**Fig 9:**
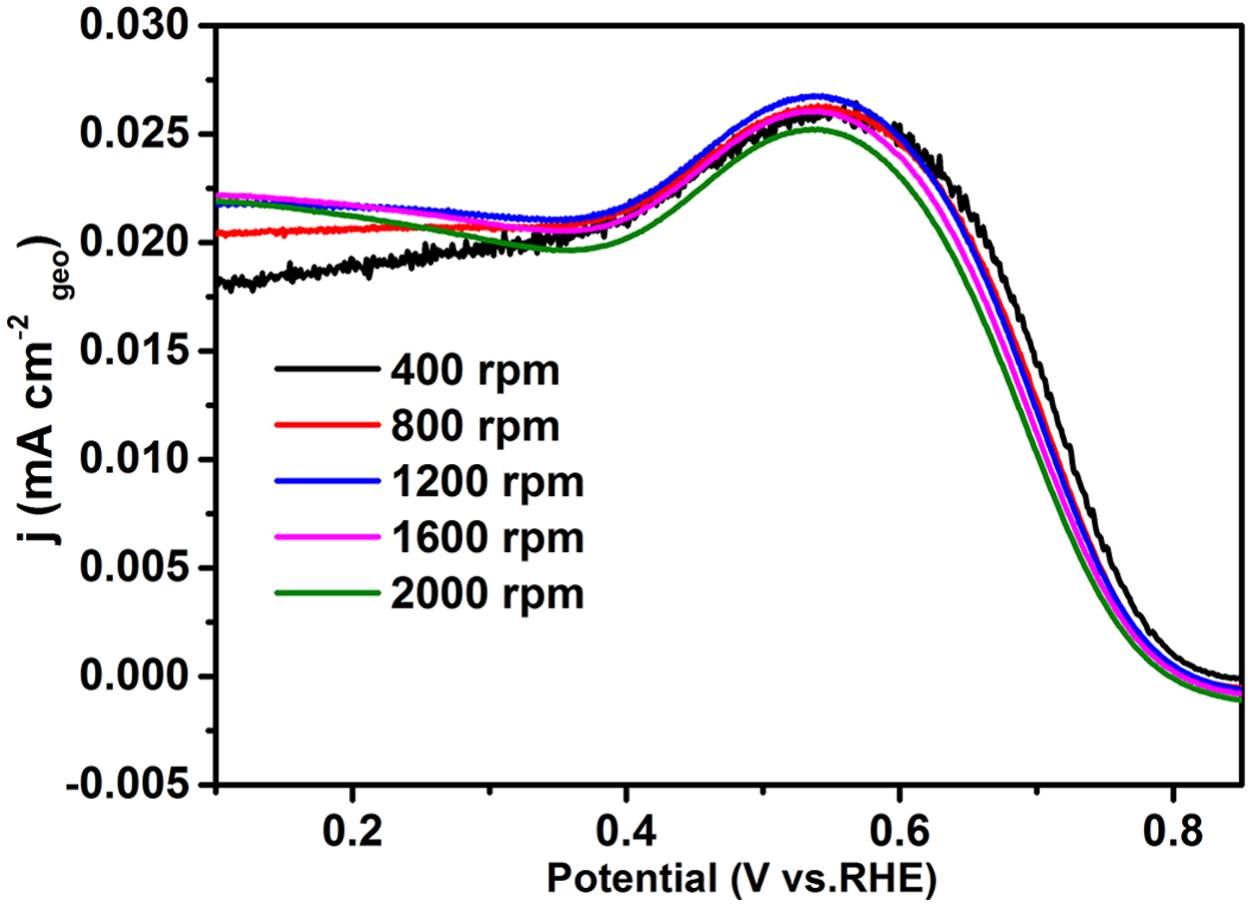
The ring current densities at different rotation rates by RRDE in O_2_-saturated 0.1 M KOH at a scan rate of 5 mV s^-1^.

Where *J* and *J*_*K*_ are the measured and kinetic-limiting current densities, *ω* is the rotation speed (rpm), *n* is the transferred electron number, *F* is the Faraday constant (*F* = 96485 C mol^-1^), *C* is the concentration of O_2_ in 0.1 M KOH solution (*C* = 1.2×10^−6^ mol cm^-3^), *D* is the diffusion coefficient of O_2_ (*D* = 1.9×10^−5^ cm^2^ s^-1^), *v* is the kinematic viscosity (*v* = 0.01 cm^2^ s^-1^). As shown in Fig 8, the numbers of electrons transferred for ORR per oxygen molecule during ORR over the potential rangefrom 0.55 to 0.10 V in the illustration increase from 2.13 to 3.21, suggesting the two steps of ORR process are the reduction of O_2_ to $HO_{2}^{-}$ and then O_2_ or $HO_{2}^{-}$ to *OH* ^−^. Moreover, the ring-disk electrochemistry has also been provided to back up the Koutecky-Levich data in Fig 9, where the oxidation current peaks located at about 0.53 V Vs. RHE can be regarded as the typical detection of $HO_{2}^{-}$ intermediate.

According to the calculations of atomic and electronic structure of molybdenum carbide phases and the adsorption and dissociation of molecular oxygen of TMCs by Illas and co-workers [45,46], β-Mo_2_C has a strong metallic character and low work function of low Miller-index surface resulting in the fact that O_2_ may either bridge two Mo atoms or attach on the top of a Mo surface atom preferably. Thus, Mo_2_C itself as well as molybdenum oxides/oxycarbide species can act as catalytic sites for the ORR after the activation of Mo_2_C. Furthermore, as a result of the approximate 2-electron reduction process for a large range of potential, G-Mo_2_C has a very possible application in the reaction of H_2_O_2_ electro-generation. However, the ORR catalytic behavior of G-Mo_2_C is inferior to the reported work by Liao [34], even comparing with the pure nanoporous molybdenum carbide wires (NP-Mo_2_C). Possible reasons can be attributed to the facile mass transport and charge transfer of NP-Mo_2_C with the aid of 1-D ordered structure and intensive nanoporous channels. Further improvement for G-Mo_2_C as ORR catalysts can be realized by supporting the nanoparticles of Mo_2_C on porous carbon materials as well as reducing the size of Mo_2_C nanoparticles.

**Fig 10:**
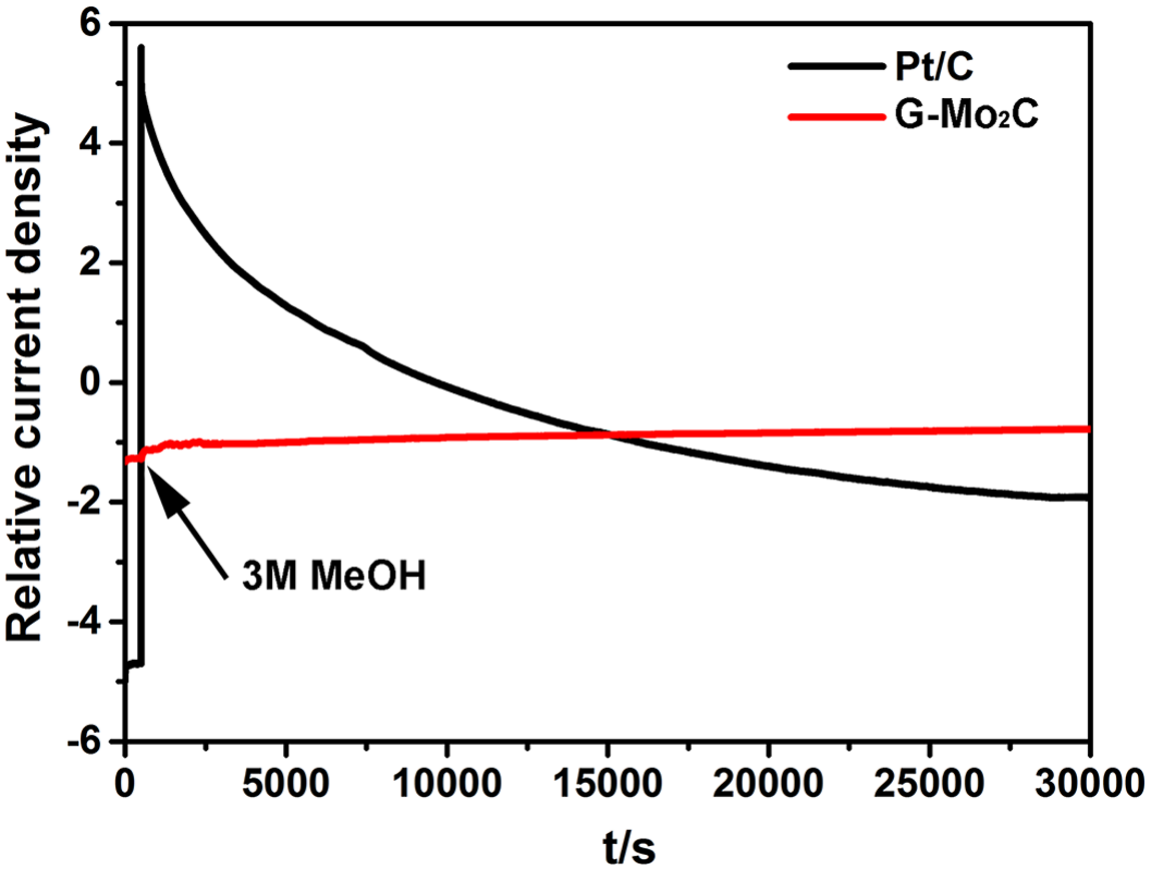
The chronoamperometric responses of Pt/C and G-Mo_2_C electrodes adding 3 Mmethanol after 500 s at 0.65 V in O_2_-saturated 0.1 M KOH at1600 rpm.

What’s more, considering the methanol crossover issue in the commercialization of alkaline direct methanol fuel cells, the chronoamperometric responses of G-Mo_2_C electrode compared with commercial Pt/C upon adding 3M methanol are shown in Fig 10. It is clear that the G-Mo_2_C electrode shows negligible change in its ORR current density after the addition of methanol at 500 s, while an instantaneous current jump is observed for Pt/C electrode contributing to the initiation of methanol oxidation reaction (MOR) [47]. The different responses demonstrate the remarkably superior methanol tolerance and high catalytic selectivity against methanol of G-Mo_2_C to commercial Pt/C as the ORR catalyst. It is believed that the superior methanol tolerance of G-Mo_2_C can be attributed to the structure and crystal phase stability of β-Mo_2_C with the addition of protective effect of graphite layers, since Mo_2_C alone hardly has electrocatalytic effect on methanol oxidation [31]. In addition, the limited degeneration of current density in the following period also indicates the good stability of as-prepared G-Mo_2_C nanocomposites.

## Conclusion

As a summary, novel graphite-Mo_2_C nanoparticles composites are synthesized by a single-step solid state reaction route. The morphology and component are further characterized by TEM, XRD and Raman, suggesting that Mo_2_C nanoparticles have a uniform distribution on the supported graphite layers. Moreover, G-Mo_2_C composites exhibit considerable electrocatalytic performances with certain activity and superior methanol tolerance for ORR in alkaline electrolyte due to the chemical interaction between the protected carbide nanoparticles and activated graphite layers.
